# Amygdalar Auditory Neurons Contribute to Self-Other Distinction during Ultrasonic Social Vocalization in Rats

**DOI:** 10.3389/fnins.2016.00399

**Published:** 2016-09-20

**Authors:** Jumpei Matsumoto, Hiroshi Nishimaru, Yusaku Takamura, Susumu Urakawa, Taketoshi Ono, Hisao Nishijo

**Affiliations:** System Emotional Science, Graduate School of Medicine and Pharmaceutical Sciences, University of ToyamaToyama, Japan

**Keywords:** auditory hallucination, self/other attribution, amygdala, ultrasonic vocalization, single unit recording

## Abstract

Although, clinical studies reported hyperactivation of the auditory system and amygdala in patients with auditory hallucinations (hearing others' but not one's own voice, independent of any external stimulus), neural mechanisms of self/other attribution is not well understood. We recorded neuronal responses in the dorsal amygdala including the lateral amygdaloid nucleus to ultrasonic vocalization (USVs) emitted by subjects and conspecifics during free social interaction in 16 adult male rats. The animals emitting the USVs were identified by EMG recordings. One-quarter of the amygdalar neurons (15/60) responded to 50 kHz calls by the subject and/or conspecifics. Among the responsive neurons, most neurons (Type-Other neurons; 73%, 11/15) responded only to calls by conspecifics but not subjects. Two Type-Self neurons (13%, 2/15) responded to calls by the subject but not those by conspecifics, although their response selectivity to subjects vs. conspecifics was lower than that of Type-Other neurons. The remaining two neurons (13%) responded to calls by both the subject and conspecifics. Furthermore, population coding of the amygdalar neurons represented distinction of subject vs. conspecific calls. The present results provide the first neurophysiological evidence that the amygdala discriminately represents affective social calls by subject and conspecifics. These findings suggest that the amygdala is an important brain region for self/other attribution. Furthermore, pathological activation of the amygdala, where Type-Other neurons predominate, could induce external misattribution of percepts of vocalization.

## Introduction

Schizophrenia is a neurocognitive disorder and auditory hallucinations are one of its most common positive symptoms (WHO, 1973). However, the neural bases of auditory hallucination are not well understood. Human MRI studies consistently report that the amygdala is an important brain region relevant to the pathology of schizophrenia: amygdala volume is reduced in schizophrenia and schizotypal personality disorder patients (Aleman and Kahn, 2005; Suzuki et al., 2005) as well as in patients with methamphetamine psychosis whose symptoms include auditory hallucinations (Orikabe et al., 2011). Furthermore, the limbic and paralimbic systems (including the amygdala) are activated when schizophrenic patients experience auditory hallucinations (Silbersweig et al., 1995; Dierks et al., 1999; Shergill et al., 2000). In epileptic patients, experiential phenomena such as perceptual hallucinations occur only when seizure discharges or electrical stimulation involve limbic structures, particularly the amygdala (Gloor et al., 1982). These findings implicate the amygdala in the generation of auditory hallucinations.

Auditory verbal communication plays important roles for mediating social interactions, and individuals have to discriminate their own speech from other individuals' speech. It has been proposed that speaking generates not only motor commands but also corollary discharges (feed-forward signals) that attenuate auditory responses to one's own overt and inner speech in the temporal cortex (Frith et al., 1998; Blakemore et al., 2000; Shergill et al., 2000; Ford and Mathalon, 2004). Auditory hallucinations are suggested to be produced by deficits in monitoring of feed-forward signals, which results in misidentification of inner speech as external voices (Ford et al., 2001; Johns et al., 2001, 2006). These hypotheses are consistent with hyper-activation of the auditory system (including the amygdala) during auditory hallucination in schizophrenic patients (Lennox et al., 2000; Northoff and Qin, 2011). However, it is unknown why and how activation of the auditory regions, especially the amygdala, leads to attribution of other individuals' agency, i.e., why auditory percepts (and accompanying activation of the amygdala) are misidentified as originating from an externally generated voice.

A possible animal model for this could be ultrasonic vocalizations (USVs) which are important for non-verbal social interaction in rats (Knutson et al., 2002; Brudzynski, 2013; Wöhr and Schwarting, 2013). Indeed, ultrasonic communication during social interaction in rodents would be useful for assessing animal models of psychiatric disorders manifesting impaired social interaction such as schizophrenia and autism (Burgdorf et al., 2013; Raza et al., 2015; Konopka and Roberts, 2016). USVs in adult rats are categorized into two types: 22 and 50 kHz calls, which are respectively associated with negative and positive affective states (Knutson et al., 2002; Brudzynski, 2013; Wöhr and Schwarting, 2013; Yuki and Okanoya, 2014). Indeed, rats use USVs for affective communications (Wöhr and Schwarting, 2013). Non-aggressive social interaction is rewarding for male rats (Douglas et al., 2004) and during this activity they reciprocally emit a number of 50 kHz calls (Brudzynski, 2013; Wöhr and Schwarting, 2013). Thus, in the present study, to investigate how the amygdala represents self (“subject”) and others' (“conspecific”) calls, we investigated the amygdalar neuronal responses to 50 kHz calls emitted during social interaction in adult male rats. The animals emitting USVs were identified by recording vocalization-related EMGs from the thyroarytenoid (TA) muscle (Riede, 2011). Based on the models of auditory hallucination evoked above, we predicted that: (1) amygdaloid neurons would respond more strongly to USVs emitted by conspecifics than those by the subject, and (2) population activity of amygdaloid neurons would represent self/other attribution of USVs.

## Materials and methods

### Animals

Thirty adult male Wistar rats weighing 270–360 g (Charles River Laboratories) and five adult female Wistar rats weighing 140–220 g (SLC, Inc.) were used. Housing temperature was maintained at 23 ± 1°C with a 12 h light/dark cycle (lights switched on at 07:00). Food and water were available *ad libitum*. The male rats were housed 2 per cage before surgery, and then were housed individually after surgery. The female rats were housed at 2 per cage throughout the experiment. All rats were treated in strict compliance with the United States Public Health Service Policy on Human Care and Use of Laboratory Animals, National Institutes of Health Guide for the Care and Use of Laboratory Animals, and Guidelines for the Care and Use of Laboratory Animals at the University of Toyama, and all experimental procedures were approved by our institutional committee for experimental animal ethics. Every attempt was made to minimize the number of animals used and their suffering.

### Surgery

Sixteen male rats (Subjects) were implanted with electrodes into the amygdala and EMG wires into the TA muscle under sodium pentobarbital anesthesia (40 mg/kg, i.p.). Electrode assemblies were implanted bilaterally aiming at the lateral amygdaloid nucleus (2.9 mm caudal from the bregma, 5.0 mm lateral from the midline, and 6.6–6.8 mm below the brain surface) based on the brain atlas of Paxinos and Watson (2006). The neuronal recording electrode assembly comprised 3 tetrodes and a microdrive. Each of the tetrodes included four tungsten microwires (20 μm in diameter; California Fine Wire) which were encased in a stainless steel cannula (30 gauge; Hakko). The wires protruded 1 mm from the tip of the cannula. The impedances of the wires were approximately 200 kΩ at 1 kHz. In addition, a bipolar stainless steel electrode (80 μm polyurethane insulated wires; the insulation was removed to expose approximately 300 μm from the tip; Unique medical) was implanted into the TA muscle according to the procedure of Riede (2011). Another 14 male rats (stimulus Conspecifics) underwent same implantation of bipolar stainless steel electrodes into the TA muscle under sodium pentobarbital anesthesia. The five female rats (stimulus Conspecifics) were devocalized by sectioning the inferior laryngeal nerve according to Nunez et al. (1985), and also ovariectomized under sodium pentobarbital anesthesia. Females are selected as control conspecifics to determine the relation between USVs and TA EMGs in the subject, in accord with the established protocol in this field (Riede, 2011, 2013, 2014).

### Experimental setup

A testing chamber (60 × 40 × 40 [height] cm) consisted of transparent acrylic was used for recording. Because ultrasound noises were emitted when rats scratched the acrylic, the floor was covered by a silicone mat (thickness = 1.5 mm). Motion of each of the interacting rats was captured and analyzed by the 3D-video based system (3D-Tracker, Matsumoto et al., 2013, 2014; Dell et al., 2014). This permitted 3D motion capture of 4 body parts (head, neck, trunk, and hip) of each rat during social interaction without applying any markers for tracking (Matsumoto et al., 2013; Dell et al., 2014). For motion capture, four depth cameras (Kinect v1 for Windows, Microsoft) surrounding the chamber captured the rats from four different viewpoints (3, 6, 9, and 12 o'clock positions; distance from the center of the chamber = 60–75 cm) and full 3D-videos were reconstructed by integrating the images captured from the four cameras. Ultrasounds were recorded by the Ultrasound Recording System (Ohara, Ltd.), which consists of a condenser microphone (TYPE7016, Aco), amplifiers, filters, an A/D converter (PCI-4461, National Instruments), and a PC for data storage. The system recorded ultrasounds ranging from 16 to 100 kHz (sampling rate = 200 kHz). The microphone was positioned 35 cm above the center of the test chamber floor. Neural activity in the amygdala and EMG signals from the TA muscle in the subject rats were amplified and transmitted by a wireless recording system (W16, Triangle Biosystems International) mounted on the head. EMGs in the stimulus conspecific were amplified and transmitted by another head-mounted wireless recording system (rodent Pack1, EMKA). The signals from both of the wireless systems were input to a common data acquisition system (OmniPlex, Plexon). The neuronal signals were digitized at a sampling rate of 40 kHz, and when waveforms crossed an experimenter-defined threshold 0.8 ms samples were stored on a computer hard disk for offline spike sorting. EMG signals from the TA muscle were digitized at a sampling rate of 1 kHz and were stored on the computer hard disk. The 3D-video recording, ultrasound recording, and neuronal and EMG recording were synchronized by a common clock signal at 30 Hz.

### Recording procedure

Prior to the first recording day, the subjects and the stimulus conspecifics were habituated to the testing chamber for 30 min. Recordings were conducted between 8:00 p.m. and 2:00 a.m. in the dark phase of the light cycle. Each day the subject was placed in the testing chamber, and neuronal activity was checked. If discriminable neuronal signals remained stable for over 10 min, a recording session was conducted. If no signal was found, the electrode assemblies were lowered by 25–100 μm and the rat was returned to its home cage.

In the recording experiment, a subject was put in the presence of, and interacted with, a stimulus male conspecific (M session) or a stimulus devocalized female conspecific (DF session) for 20 and 5 min, respectively. At the end of DF sessions, recordings continued for 5 min while the subject was alone. These sessions all occurred in the same testing chamber. One M session and one DF session were conducted for each subject on the same day. In addition, a DF session was also conducted for each of the stimulus male rats studied that day. Neuronal activity and TA EMG from the subject rat, TA EMG from the stimulus male rat, ultrasounds, and 3D-videos were recorded during the M session. In the DF session, the recordings were performed similarly with TA EMG recording from the devocalized stimulus female rat. To reduce potential variations in social behavior related to differences in body sizes (e.g., Wesson, 2013), pairs of subject and stimulus male conspecifics were chosen so that the conspecific's body weight varied less than ±20% from that of the subject. After the recording session, the electrode assembly was lowered by at least 100 μm to record new neuron(s) for the next session.

### Data analysis: USV detection and assignment

Each call of USVs was automatically detected with custom written MATLAB scripts (Mathworks) implementing an algorithm adapted from Reno et al. (2013). First, a sonogram (2.5 ms time window, 0.5 ms time step, 0.4 kHz bandwidth) of a recorded ultrasound was calculated. The power at each step of the sonogram was converted into z-scores, normalized relative to the baseline level at each frequency during the first 1 s silent period from the onset of the recording. In the sonogram, a cluster of pixels (pixel size = 0.5 ms × 0.4 kHz) with Z > 2.5 including at least one pixel with Z > 3.0 was considered as a call. For detecting the 50 kHz calls, the clusters within the 30–100 kHz range were examined. If the pixels located with intervals of < 20 ms in the time axis and < 40 kHz in the frequency axis, the pixels were considered to belong to a cluster (call). Clusters briefer than 5 ms were ignored. The 22 kHz calls were similarly detected by searching within the 19–30 kHz range. For the 22 kHz calls, the intervals of pixels in a cluster were set as < 100 ms on the time axis and < 8 kHz on the frequency axis, and clusters briefer than 100 ms were ignored. In addition, noise removal algorithms were implemented for removing harmonics of 22 kHz calls (Reno et al., 2013) and removing noise with high power in a low frequency band (16–30 kHz; Sirotin et al., 2014). Finally, for each of the calls detected, we calculated timings of the onset and the offset, the minimum and the maximum frequencies and the frequency modulation range (FM range, i.e., the maximum frequency minus the minimum frequency). Furthermore, the following acoustic parameters of each call were calculated following the work of Yuki and Okanoya (2014). (1) mean amplitude (instantaneous amplitude of the sound calculated after Hilbert transformation), (2) maximum amplitude, (3) latency from onset to maximum amplitude (“latency for max” in **Table 2**), (4) maximum harmonics-to-noise ratio (“max HNR”), (5) number of sub-elements within a call, (6) number of frequency modulations (number of vertical peaks and valleys in the sonogram; “No. of FM”), (7) total frequency modulation (mean frequency modulation per millisecond within a call; “total FM”), (8) bandwidth in the center (the midpoint between the start and end of the call) of a call (“BW in center”), (9) bandwidth increase from the start to the center of a call (“BW center–start”), (10) bandwidth increase from the center to the end in a call (“BW end–center”), and (11) duration of silent period before onset of a call (“interval before onset”).

EMG signals from the TA muscle were bandpass-filtered (100–300 Hz) and the instantaneous amplitude of the signal was calculated from Hilbert transformation, using the *hilbert* routine of MATLAB (Mathworks). For normalization, the EMG amplitude at each time point was divided by the standard deviation of the EMG amplitude in the periods without USVs, resulting in a signal-to-noise (S/N) ratio of the amplitude. To analyze correlations between the EMG amplitude and 50 kHz calls, the maximum amplitude from 20 ms prior to the onset of each call to 20 ms after the end of the call was calculated. The correlation analysis of 50 kHz calls during DF sessions revealed that 50 kHz calls involving wide range frequency modulation (>15 kHz, WFM calls) were almost always accompanied high maximum EMG amplitude (S/N > 3.0; see Results for details, Figure 2). Therefore, each WFM call during an interaction with a stimulus male rat was assigned to one of the two rats based on the EMG amplitude, as follows. When the maximum EMG amplitude of the subject was >3.0 while that of the stimulus conspecific was < 3.0, the WFM call was assigned to the subject. When the maximum EMG amplitude of the subject was < 3.0 while that of the stimulus conspecific was >3.0, the WFM call was assigned to the stimulus rat. In the remaining cases (if the maximum EMG amplitude of subject and stimulus rats were both >3.0 or < 3.0), the WFM call was not assigned and was not used for further analyses.

### Spike sorting

**Figure 1:**
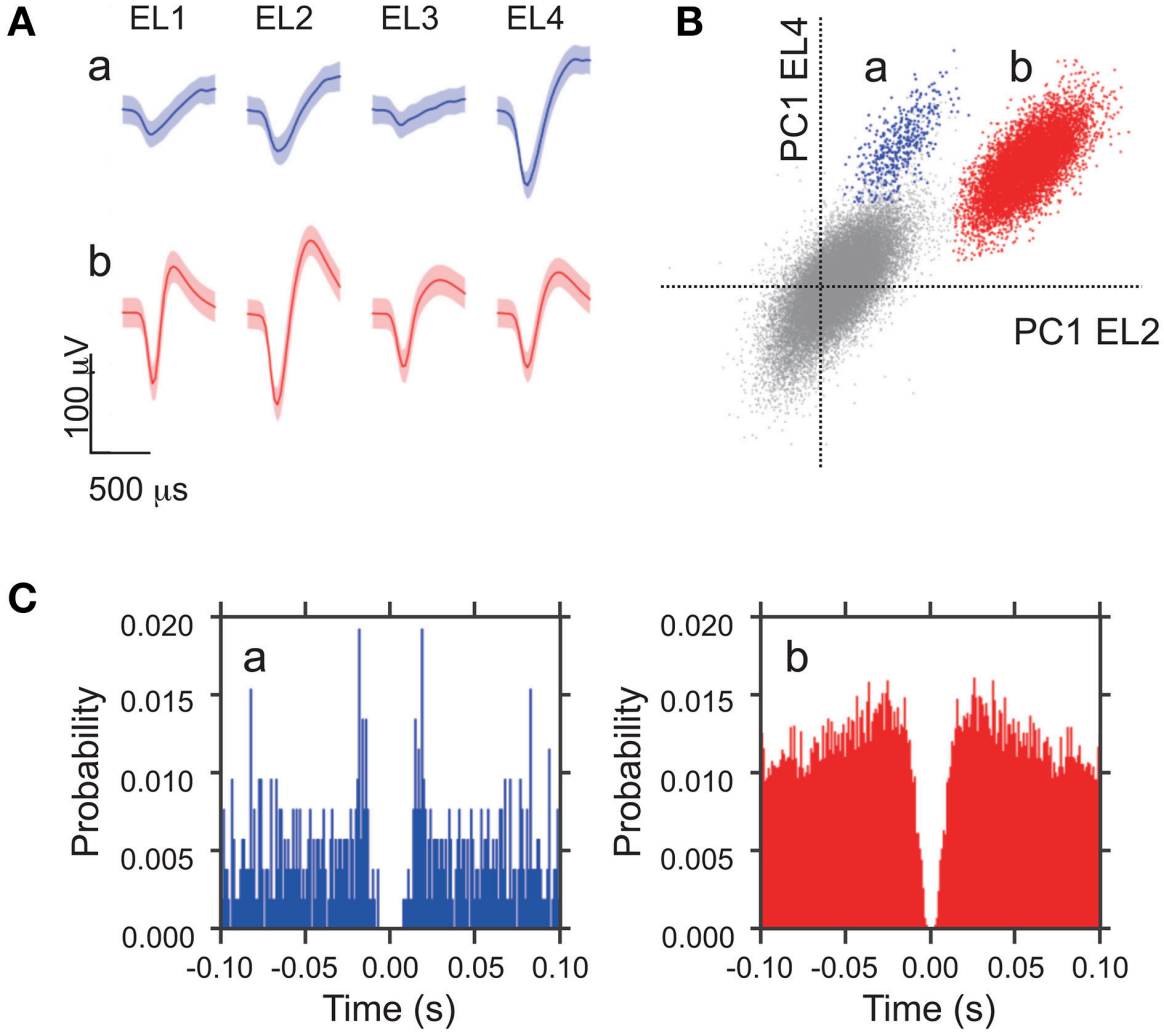
**Waveform characteristics of two representative amygdala neurons. (A)** Waveforms (mean ± SD, shaded) simultaneously recorded from the four tetrode leads (EL 1–4). The waveforms indicated by a and b correspond to the two clusters in **(B)**. **(B)** The results of an offline cluster analysis. Each dot represents one spike. The horizontal axis represents the first principle component (PC 1) of EL 2, and the vertical axis represents PC 1 of EL 4. **(C)** Autocorrelograms of neurons a and b. Autocorrelograms of neurons a and b show that refractory periods of the neurons were greater than 2 ms, consistent with these spikes originating from single neurons. Bin width = 1 ms.

Digitized neuronal activity was discriminated into single units according to waveform components with the Offline Sorter^TM^ program (Plexon). Briefly, each of the recorded waveforms was plotted in two- or three-dimensional feature spaces; various features of spike waveforms (waveform projection onto principal components, peak amplitudes of the waveforms, valley amplitudes of the waveforms, peak-valley amplitudes of the waveforms, etc.) were selected as a dimension. Spikes in each cluster in the feature space were considered as a single unit if they passed the following four criteria: (1) the cluster boundaries were well separated from the other clusters; (2) waveform shapes in the cluster were consistent; (3) the waveform shapes were consistent with those of action potentials; (4) an absolute refractory period of at least 1.0 ms was observed in an interspike interval histogram. The isolated single units were then transferred to the NeuroExplorer® program (Nex Technology) for further analysis. Typically, 1–4 single units were isolated by offline cluster analysis from the four channels (wires) of a single tetrode (see Figure 1).

### Analysis of correlation between neural activity and assigned USVs

If activity from the same neuron was recorded in more than five WFM calls by a rat during a M session, the data were analyzed as follows. Four periods around the onset of the USVs were defined: baseline (−80 to −40 ms), PRE1 (−40 to 0 ms), POST1 (0 to 40 ms), and POST2 (40 to 80 ms). Neural firing rates were compared among these 4 periods using the Friedman test and if this result was significant (*p* < 0.05), the neuron was considered to be responsive to the vocalization. Significant excitatory or inhibitory responses during each period of USVs were defined by a Wilcoxon signed rank test with Bonferroni correction (*p* < 0.05) of neuronal activity between the baseline period and other periods. Responsive neurons without significant Wilcoxon test results were categorized as “unclassified.” No neurons showed both excitatory and inhibitory responses. For each excitatory or inhibitory neuron, response magnitude and latency were measured. The response latency was defined as the center of the earliest period that showed a significant difference. The response magnitude was defined as the largest difference in firing rates between the baseline and other periods.

Neural responses to WFM calls by the stimulus male conspecific and the subject rat were separated according to the method described above and analyzed. Neurons showing significant responses to the WFM calls only from the stimulus rat were categorized as Type-Other neurons. Neurons showing significant responses to the WFM calls from subject-initiated vocalization were called Type-Self neurons. Neurons showing significant responses to the WFM calls by both the subject and conspecific were categorized as Type-Both neurons. To assess response selectivity of Type-Other and Type-Self neurons, a selectivity index (*SI*) was calculated for each neuron. *SI* was defined as *SI* = *M*_*pref*_ / (*M*_*Conspecific*_ + *M*_*Subject*_); where *M*_*Conspecific*_ and *M*_*Subject*_ represent response magnitudes to calls by conspecific and subject, respectively, while *M*_*pref*_ represents *M*_*Conspecific*_ and *M*_*Subject*_ in Type-Other and Type-Self neurons, respectively.

To assess population coding of self-other selective activity, the population firing pattern (*PFP*) of all the responsive neurons to calls by subject and by conspecifics was calculated for each of the four periods using the vector *PFP* = (*F*_**1**_, *F*_**2**_, …, *F*_*n*_); where *n* represents the number of responsive neurons and *F*_*i*_ represents normalized firing rate of *i*-th responsive neuron in a given period (Young and Yamane, 1992; Kiani et al., 2007). The normalized firing rate (F) of a neuron in a given period was calculated as follows: F = (f – f_min_) / (f_max_ – f_min_); where f is the firing rate of the period, f_max_ and f_min_ are maximum and minimum firing rates among the four periods of the calls by the subject or conspecific.

### Histology

After the experiments, all subject rats were deeply anesthetized with sodium pentobarbital (50 mg/kg, i.p.) and a 20-μA cathodal current was applied through the recording electrodes for 30 s to make a small electric lesion at the tip of each tetrode. The subject rats were then transcardially perfused with 0.9% saline followed by 10% buffered formalin containing 2% potassium ferricyanide. The brain was removed and fixed in 10% formalin for at least 48 h. Serial sections of 50 μm were cut on a freezing microtome and stained with Cresyl Violet. Electrode locations were verified microscopically (Supplementary Figure 1) and identified with reference to the atlas of Paxinos and Watson (2006).

## Results

### Assignment of USVs based on EMGs

**Figure 2:**
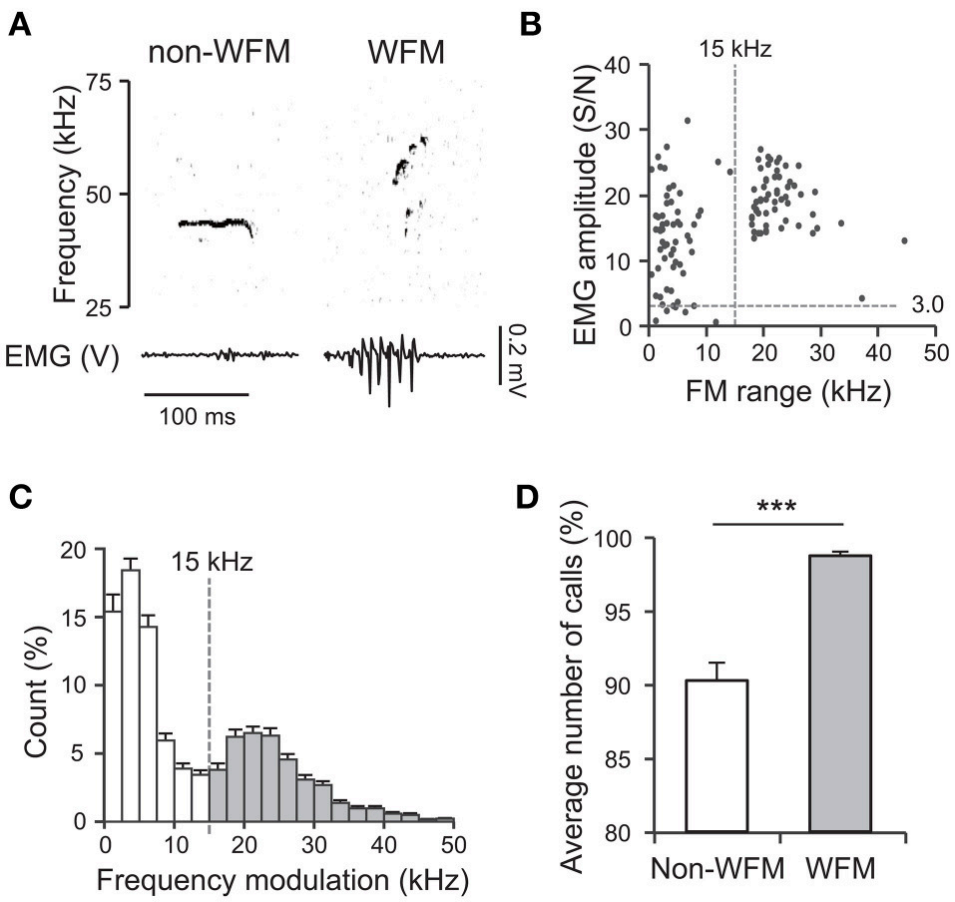
**Fifty kilohertz calls and TA EMG. (A)** Examples of simultaneously recorded sonograms and TA EMGs acquired during recording sessions where subjects interacted with devocalized female rats (DF sessions) [left: a call with limited range frequency modulation (non-WFM call); right; a call with wide range frequency modulation (WFM call)]. **(B)** In a representative DF session, the relation between the range of frequency modulation (FM range) of 50-kHz calls and the maximum EMG amplitude around the calls. Each point represents a call. **(C)** The distribution of frequency modulation ranges of high frequency calls of all DF sessions. White bars: calls with frequency modulation < 15 kHz (non-WFM calls); gray bars: calls with frequency modulation >15 kHz (WFM calls). Error bar: SEM. **(D)** The percentage of all non-WFM and WFM calls emitted with clear EMG activity (maximum amplitude >3) over all DF sessions. Error bar: SEM. ^***^ signifies *p* < 0.001, paired *t*-test.

To determine the relation between USVs and TA EMGs, first we analyzed the data acquired when subjects interacted with devocalized female rats (DF session). The data from 50 sessions with 30 male rats (16 subjects and 14 stimulus female rats; 1–4 sessions for each rat) were analyzed. During the DF sessions, the mean number of 50 kHz calls per 10 min session was 239.1 ± 30.7 (SEM; range 13–1164). Almost no 22 kHz calls were observed (only once in all 50 sessions). Figure 2A shows examples of 50 kHz calls and TA EMGs simultaneously recorded from a subject male rat. The 50 kHz call with clear frequency modulation (Figure 2A, right) was accompanied by strong TA muscle activity (S/N > 3.0), while the 50-kHz call with low frequency modulation (Figure 2A, left) was not. Figure 2B shows the relation between the range of frequency modulation (FM range) of 50 kHz calls and the maximum EMG amplitude during the calls in different trials. The results indicated that 50 kHz calls with wide range frequency modulation always corresponded to strong TA muscle activity. In addition, there seemed to be two clusters in the distributions in the FM range (Figure 2B). By tallying the incidence of the different FM ranges of 50 kHz calls across the 50 sessions, we confirmed that there are two clusters separated by a border at 15 kHz (Figure 2C). Based on these results, we defined the 50 kHz calls with FM range >15 kHz (Figure 2C, gray bars) as wide range frequency modulated calls (WFM calls). Then, we compared incidences of WFM (98.7 ± 0.3%) and non-WFM calls (90.2 ± 1.2%) accompanied by high-amplitude EMG activity (Figure 2D). The results showed that more calls with high-amplitude EMG activity were WFM than non-WFM (paired *t*-test, *p* = 1.8 × 10^−9^). In addition, the high incidence (98.7%) of WFM calls accompanying high-amplitude EMG activity suggests that WFM calls were almost always accompanied with high-amplitude TA EMGs.

**Figure 3:**
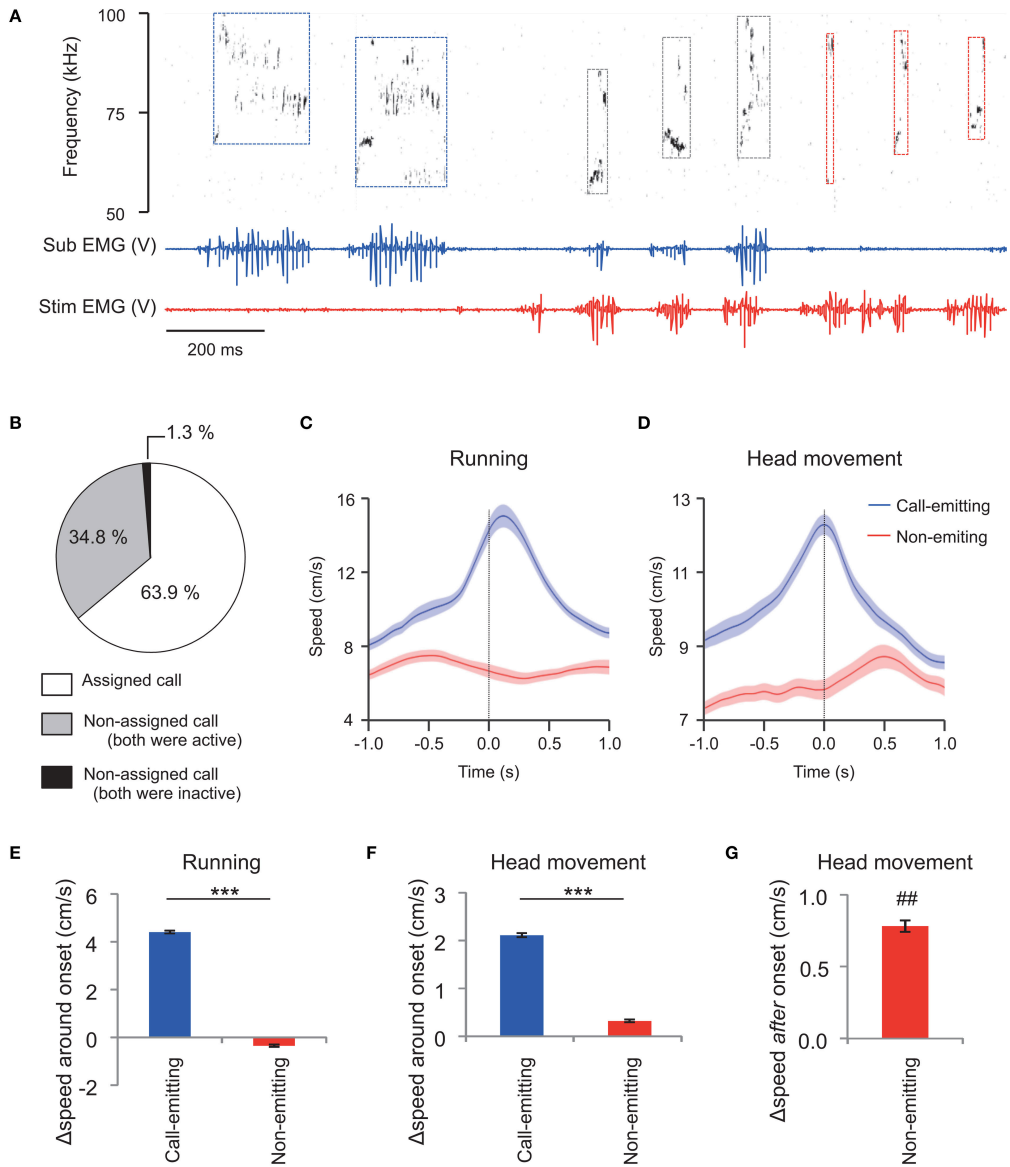
**Assignment of WFM calls during social interactions between vocalizing male rats (M sessions). (A)** An example of call assignment. Simultaneously recorded sonogram (top), TA EMG of a subject rat (middle), and TA EMG of a stimulus conspecific (bottom) are shown. Each rectangle demarcated by dashed lines in the sonogram represents one call and the color of the square represents estimated source of the call (blue: the subject rat; red: the stimulus rat; black: indistinguishable) based on the concurrent TA EMG. Sub: subject; Stim: stimulus rat. **(B)** Distribution of call assignments. **(C,D)** Averaged (horizontal plane) speeds measured from the trunks **(C)** of and the head (translation) relative to the trunk **(D)**. Speeds are shown for the call-emitting (blue) and non-emitting (red) rats. The solid lines and translucent areas indicate the means and SEMs, respectively. Time zero represents the onset of the calls. **(E,F)** The change in trunk speed in horizontal plane and head movement relative to the trunk around the onset of WFM calls [Δspeed around onset = (mean speed during the first 0.5 s after call onset) − (mean speed at −1.0 to −0.5 s prior to call onset)] of either the call-emitting (blue) or non-emitting (red) rats. (^***^ indicates *p* < 0.001, paired *t*-test). **(G)** The change in head movement relative to trunk after the onset of WFM calls [Δspeed after onset = (mean speed at 0.35–0.45 s after call onset) − (mean speed at −0.1–0.0 s prior to call onset)] of the non-emitting rats. (## signifies *p* < 0.01, one-sample *t*-test).

Based on these results, during exposure of the subject to the stimulus male conspecific, we assigned each WFM call to the rats that had high-amplitude TA EMG activity (see Materials and Methods for the detail). During social interaction, rats emitted frequent ultrasonic vocalizations (about 65 calls/min on average), and also had active physical contact (See Supplementary Movie 1 as an example). Figure 3A shows an example of assignments of WFM calls.

In total, the assigned WFM calls were obtained from 27 sessions of interactions between the subject and male stimulus rats (M sessions). During these 20 min sessions, there was a mean of 1325.2 ± 170.2 ultrasound calls (range 160–3816). The incidence of WFM calls among all ultrasound calls was 46.4 ± 2.1%. Among the WFM calls, the incidence of the assigned alls was 63.9 ± 2.2% (Figure 3B, white area). Thus, around 30% of all ultrasound calls could be assigned using TA EMGs. Among all WFM calls, only 1.3 ± 0.5% showed no measurable high-amplitude EMGs in either of the two rats (Figure 3B, black area), confirming that WFM calls almost always accompanied these high-amplitude EMGs. Since previous studies (Barfield et al., 1979; Himmler et al., 2014) reported that 50 kHz calls were often associated with motion of the rats, we calculated mean running speeds (derived from horizontal trunk positions) and head movement (speed of the head relative to the trunk) of call-emitting and non-emitting rats around the onset of WFM calls (Figures 3C,D). The change of the speed of the call-emitting rats around the onset of WFM calls was significantly greater than those of the non-emitting rats (Figures 3E,F; paired *t*-test, *p* < 0.001), consistent with previous reports. The results further confirmed the validity of the assignment based on EMGs. Interestingly, the change of speed of head movements of the non-emitting rats after the subject call onsets was significantly positive (Figure 3G; one-sample *t*-test, *p* = 0.0095), indicating that the non-emitting rats responded to the calls.

### Amygdala neural response to the assigned calls

**Figure 4:**
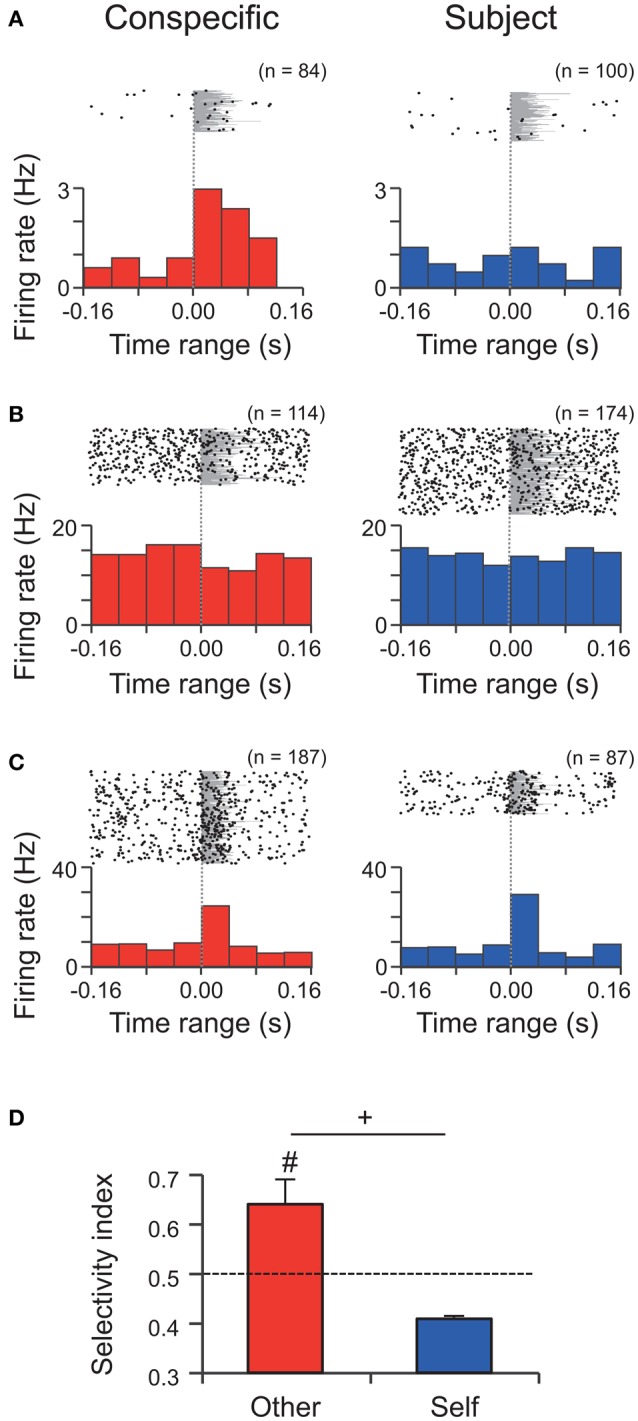
**Single unit activity during WFM calls by the conspecific and the subject in M sessions. (A–C)** Perievent rasters and histograms of three neurons **(A)** an excitatory Type-Other neuron; **(B)** an inhibitory Type-Other neuron; **(C)** an excitatory Type-Both neuron. Bin widths = 40 ms. Time zero represents onset of the call. Each number (n) at the top right of the panel indicates the number of calls analyzed. Gray shading indicates the call durations. **(D)** The response selectivity (selectivity index, SI) of Type-Other and Type-Self neurons. The dashed line indicates the expected value if responses to the WFM calls by the conspecific and subject were equal. #, significantly different from the expected value, *p* < 0.05, Wilcoxon signed rank test; +, tendency of the difference between Type-Other and Type-Self neurons, *p* < 0.1, Wilcoxon rank sum test.

A total of 60 amygdalar neurons were recorded from 16 rats. Figure 4 shows examples of three types of neuronal responses. Type-Other neurons showed excitatory and inhibitory responses to WFM calls by the conspecific (Figures 4A,B), while Type-Both neurons showed excitatory responses to WFM calls by both the subject and the conspecific (Figure 4C). The numbers of neurons showing each pattern of responses are tallied in Table 1. The average response latencies among all excitatory and inhibitory responding neurons were 27.3 ± 10.6 ms (*n* = 13). The average response latencies in Type-Other, Type-Self, and Type-Both neurons were 30.0 ± 12.5 ms (*n* = 8), 20.0 ms (*n* = 1), and 20.0 ms (*n* = 2), respectively.

**Table 1:** **Incidence of each type of amygdalar neurons with excitatory or inhibitory responses**.

	**Excitatory**	**Inhibitory**	**Unclassified**	**Total**
Type-Other	6	2	3	11
Type-Self	1	0	1	2
Type-Both	2	0	0	2
Total	9	2	4	15

Type-Other: Neurons showed significant response to the calls by the conspecific but not to those by subject. Type-Self: Neurons showed significant response to the calls by subject but not to those by the conspecific. Type-Both: Neurons showed significant response to the calls by both of subject and the conspecific.

A total of 15 neurons (25%) responded to WFM calls. Most of these neurons responded to the calls by conspecifics but not subjects (Type-Other, 11 neurons, 73% of the responsive neurons). The incidences of the three types of responses (Type-Other, Type-Self, and Type-Both) were not equal (chi-square test, *p* = 0.0045). The *post-hoc* residual analysis revealed that the ratio of Type-Other neurons were significantly larger than the average (*p* = 0.0073), indicating that most neurons in our sample are selective to the WFM calls by the conspecifics. The selectivity of the responses of Type-Other neurons (*SI* = 0.64 ± 0.05) was significantly higher than the expected value assuming that the responses to calls by conspecifics and subjects would be equal (*SI* = 0.5; Wilcoxon signed rank test, *p* = 0.0127; Figure 4D left), while the selectivity of the Type-Self neuron (*SI* = 0.41 ± 0.01) was not different from the expected value (Wilcoxon signed rank test, *p* = 0.5; Figure 4D right). In addition, the selectivity index of Type-Other neurons tended to be higher than that of Type-Self neurons (Wilcoxon rank sum test, *p* = 0.0769; Figure 4D). These results suggest that the amygdala preferentially responds to the WFM calls by conspecifics.

To investigate population coding of self-other attribution in this sample, population firing patterns (PFP, see Materials and Methods) were analyzed. PFP of all neurons responsive to conspecific calls was significantly different from PFP for subject calls during the periods 0–40 and 40–80 ms after the onset of the calls (Pearson's correlation coefficients, *r* = −0.02 (*p* = 0.94), and *r* = 0.02 (*p* = 0.94) during 0–40 and the 40–80 ms periods, respectively). This strongly suggests that these neurons are involved in self-other attribution.

Acoustic features of 50 kHz calls can vary depending on different affective contexts (Yuki and Okanoya, 2014) and the selective response of Type-Other neurons could have been confounded by differences between acoustic features of 50 kHz calls emitted by subjects vs conspecifics. To investigate this, we compared various acoustic parameters of WFM calls in M sessions where Type-Other neurons were recorded (Table 2). The results indicated that there were no significant differences in any of the acoustic features between WFM calls by subject and conspecific (*p* > 0.05, paired *t*-test). This indicates that the selective responses of Type-Other neurons could not be ascribed to differences in the acoustic features of the calls.

**Table 2:** **Comparisons of mean auditory feature parameters of WFM calls from the subject and the conspecific**.

	**Subject**	**Conspecific**	***p*-value**
Duration (ms)	38.1 ± 2.9	36.2 ± 2.6	0.67
FM range (kHz)	25.1 ± 0.8	23.9 ± 0.7	0.34
Mean amplitude (dB)	−37.6 ± 0.2	−37.4 ± 0.2	0.40
Max amplitude (dB)	−23.0 ± 0.2	−22.9 ± 0.2	0.87
Latency for max (ms)	17.9 ± 1.5	16.2 ± 1.3	0.34
Max HNR	3.72 ± 0.06	3.83 ± 0.04	0.20
No. of sub-elements	3.09 ± 0.19	2.90 ± 0.17	0.40
No. of FM (/ms)	0.134 ± 0.003	0.151 ± 0.009	0.16
Total FM (kHz/ms)	1.38 ± 0.09	1.51 ± 0.07	0.23
BW in center (kHz)	2.06 ± 0.25	2.38 ± 0.28	0.46
BW center–start (kHz)	0.98 ± 0.26	1.40 ± 0.28	0.34
BW end–center (kHz)	−1.30 ± 0.24	−1.48 ± 0.21	0.55
Interval before onset (s)	1.72 ± 0.47	1.26 ± 0.24	0.18

The data was obtained from M session where Type-Other neurons were recorded (n = 10). The data is represented as mean ± SEM. The p-values were computed from paired t-tests between the parameters of the subject and the conspecific in the same sessions. See Materials and Methods for definitions of the parameters. dB, decibels relative to the maximal range of recording.

**Figure 5:**
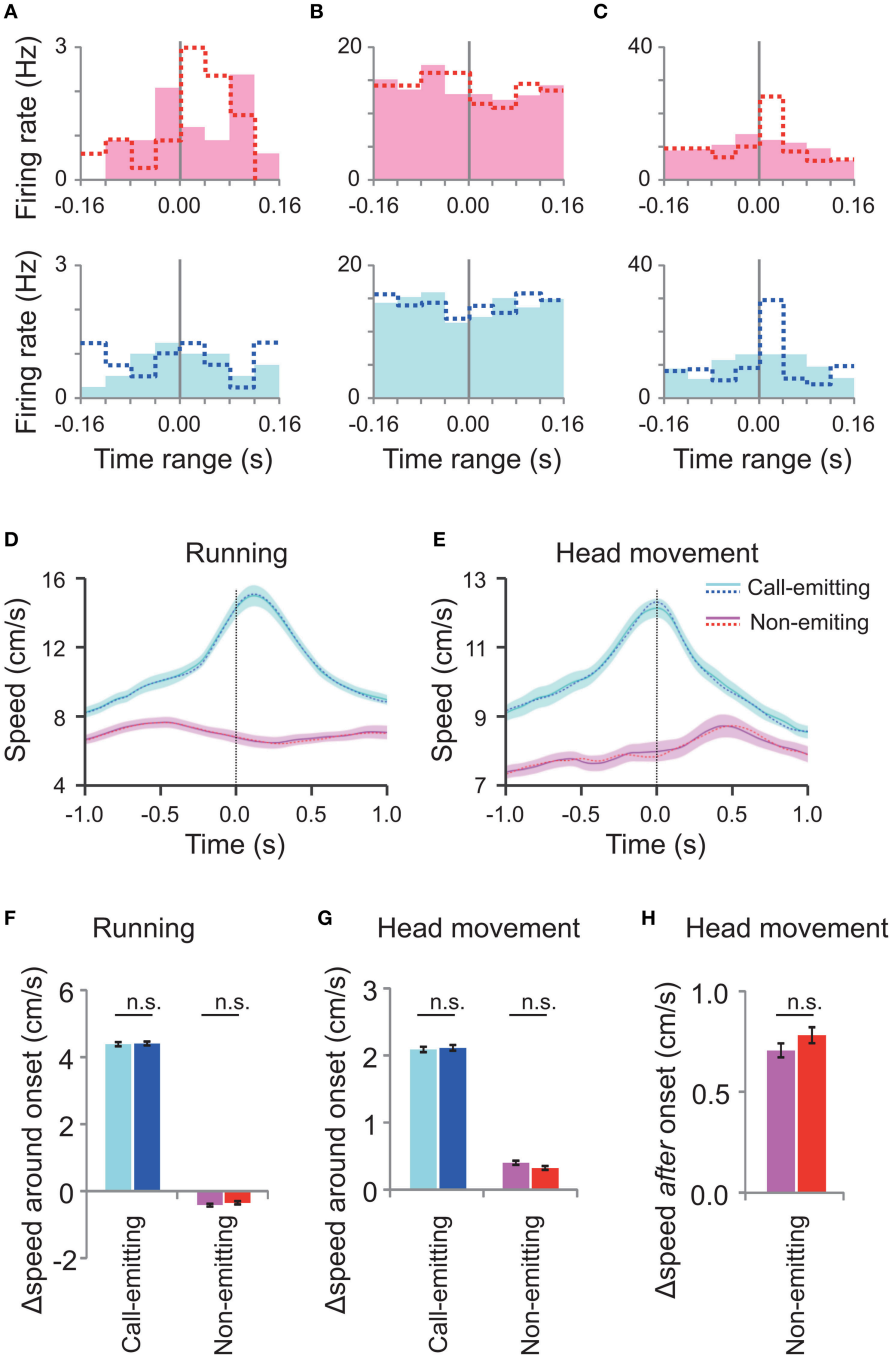
**Neuronal activity and rats' motion around the randomly shifted call onsets. (A–C)** Peri-event histograms of spike activity around the randomly shifted call onsets (filled bars) of neurons shown in Figures 4A–C, respectively. Dotted lines indicate original responses. Responses to the call by the conspecific (pink) are displayed above and those of the subject (blue) below. **(D,E)** Average running **(D)** and head movement **(E)** speeds of interacting male subject and conspecific rats (30 rats; 27 M sessions) around the randomly shifted call onsets by the call-emitting (light blue) and non-emitting (pink) rats and the original data before the random shift (dotted line). The other descriptions are the same as those for Figures 3C,D. **(F,G)** The change in running speed and head movement around the randomly shifted onsets (left; light blue and pink) and original ones (right; blue and red) of either the call-emitting (light blue/blue) or non-emitting (pink/red) rats. Note that the speeds at the different onsets showed no significant difference (paired *t*-test, *p* > 0.05). Other descriptions are the same as those for Figures 3E,F. **(H)** Comparison of head movement after the random-shifted onsets (left) and original ones (right) of the non-emitting rats. The speed changes for the different onsets were not significantly different (paired *t*-test between the data before and after the random shift, *p* > 0.05). Other descriptions are the same as those for Figure 3G.

Although, we focused on the very short time range (±80 ms) around the call onset, there was concern that the above neural correlates with the call were not auditory responses but rather neural responses to concurrent motions of rats associated with the call (Figures 3C–G). To investigate this issue, we generated randomly-shifted call onsets by slightly (within −80 ms to +80 ms) shifting the original WFM call onsets. Almost all (93%, 14/15) of the responsive neurons did not show any significant difference of the activity around the random-shifted call onsets (Friedman test, *p* > 0.05; Figures 5A–C), indicating that their responses were indeed time-locked to the call onset. On the other hand, random shifts of the rat movement data (as above) did not significantly change the profile of the motions of rats around the call onsets compared with the original data (Figures 5D–H). Thus, these analyses indicate that the call-related neural responses were not associated with the motions of rats but rather with the other signals related to the calls.

We found that subject's average head movement speed increased after a WFM call was emitted by the conspecific (Figures 3D,G). Thus, the Type-Other neuron activity may be related to behavioral responses to the calls. To test this hypothesis, we first examined the correlation between the neuronal response magnitudes and the head movements after each of the WFM calls by conspecifics. One of the Type-Other neurons showed the significant positive correlation (*p* = 0.0414, Spearman's correlation analysis; Supplementary Figure 2). Randomly shifting the call onsets extinguished this correlation (*p* = 0.900, Spearman's correlation analysis), indicating the correlation was attributed to the neural responses to the auditory signals of the calls rather than the associated motion. Taken together, these results are more consistent with Type-Other neuronal responses contributing to behavioral reaction to the calls by conspecifics than the converse.

**Figure 6:**
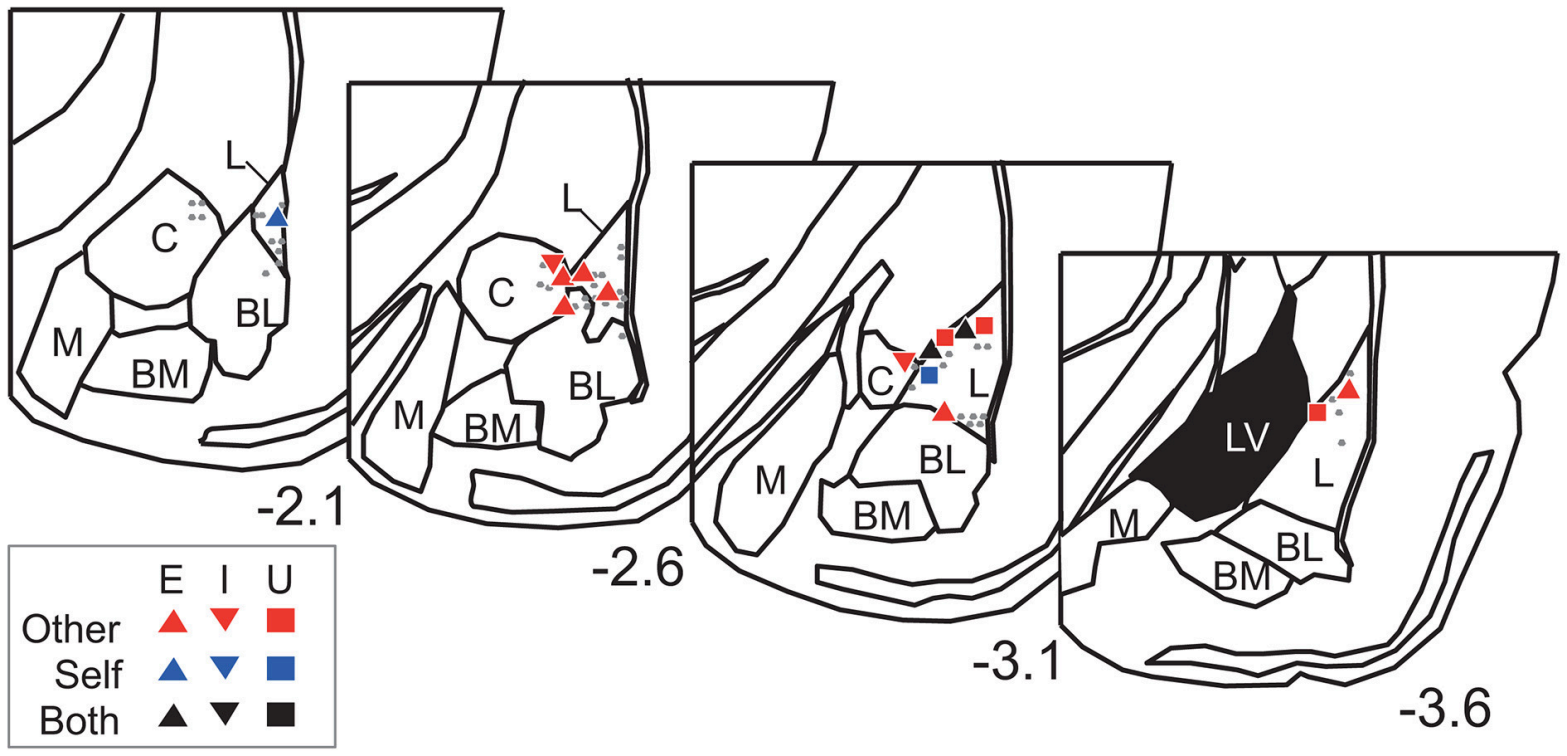
**Recording site histological analyses**. Positions of Type-Other, Type-Self, and Type-Both neurons are represented by red, blue, and black symbols, respectively. The inset keys indicate response types. Gray dots represent positions of non-responsive neurons. The value below each section indicates distance (mm) from bregma. L, lateral amygdaloid nucleus; BL, basolateral amygdaloid nucleus; BM, basomedial amygdaloid nucleus; M, medial amygdaloid nucleus; C, central amygdaloid nucleus; LV, lateral ventricle. The atlas diagrams were adapted from Paxinos and Watson (2006) with permission.

The neurons were recorded from the dorsal part of the amygdala, particularly from the lateral amygdaloid nucleus (Figure 6). Overall, these results suggest that dorsal amygdala neurons process others' vocalizations and distinguish them from those made by the subject.

## Discussion

One quarter of the amygdalar neurons (15/60) responded to 50 kHz calls emitted by subjects and/or conspecifics. Among the responsive neurons, most neurons (Type-Other neurons) (73%, 11/15) responded to calls by conspecifics but not those by subjects. Although, two neurons (Type-Self neurons; 13%, 2/15) responded to calls by subjects but not those by conspecifics, response selectivity of these neurons was lower than those of Type-Other neurons. The remaining neurons (13%, 2/15) responded to both calls by subjects and conspecifics. These auditory responsive neurons were located in the dorsal amygdala including the lateral amygdaloid nucleus that receives auditory inputs from other structures (Duvarci and Pare, 2014). The present results provide the first neurophysiological evidence that the amygdala discriminatively represents affective social calls by self from others since both types of calls had indistinguishable acoustic characteristics. Furthermore, population coding of amygdalar neurons represented distinction of calls made by self vs. others. These findings support a role for the amygdala in self/other discrimination.

### Comparison with the previous studies

The auditory cortex sends signals to the amygdala (Romanski and LeDoux, 1992) and auditory cortical neurons respond differently to vocalization by subjects or conspecifics in monkeys and humans (Müller-Preuss and Ploog, 1981; Creutzfeldt et al., 1989a,b; Eliades and Wang, 2003). These findings are consistent with the present results although there are some differences. First, activity of most neurons was inhibited in response to self-vocalization in the auditory cortex (Eliades and Wang, 2003), while here the amygdalar neurons (Type-Self and Type-Both neurons) showed excitatory responses to the rat's own calls. Second, a previous study reported most amygdalar neurons in rats showed inhibitory responses to playback of the 50 kHz calls by conspecifics when the subject was alone in a test chamber (Parsana et al., 2012), while almost all auditory cortical neurons in monkeys showed excitatory responses to playback of calls when the subject was alone in a chamber (Müller-Preuss and Ploog, 1981; Eliades and Wang, 2003). In the present study, amygdalar neurons showed only excitatory responses to calls by actual conspecific rats, not recordings.

Indeed none of the previous studies examined single neuronal responses to calls or voices during active social interaction. Thus, some new observations here are likely related to being in the company of and interacting with another rat. Here, most amygdalar neurons showed excitatory responses to the 50 kHz calls emitted from an actively interacting partner in contrast to the previous playback experiment (Müller-Preuss and Ploog, 1981; Eliades and Wang, 2003; Parsana et al., 2012). The 50 kHz calls by the conspecifics were associated with the conspecific's motor activity with relation to the subject (Figures 3C–F). During social interaction, the subject may need to quickly react to the conspecific's calls, adding social behavioral relevance to the calls as opposed to the calls played back from a speaker in the previous studies (Müller-Preuss and Ploog, 1981; Eliades and Wang, 2003; Parsana et al., 2012). This may be an important factor in the activation of the amygdalar neurons found here, because the amygdala has been suggested to process the behavioral relevance of stimuli (Fitzgerald et al., 2006; Adolphs, 2008, 2010). Consistent with this hypothesis, the response of a Type-Other neuron (1/11) correlated with the call that-preceded the subject's head movement, (Figure 6) consistent with the notion that the amygdalar neural response contributed to the behavioral response to the calls. Further work will be necessary to confirm this. Further studies should also directly compare responses of the same neurons to the same calls in the presence and absence of the call-emitting conspecific. Taken together, these results underline the importance of studying neural responses to social signals in natural interactive situations (Redcay et al., 2010) to understand functions of the amygdala.

### Possible neural mechanisms of pathological auditory hallucinations

This experimental model could help shed light on developing experimental models for auditory hallucinations in schizophrenia. Here, most responsive neurons in amygdala (73%, 11/15) responded strongly to calls by conspecifics and population activity was sensitive to the self/other distinction. These results are consistent with the idea that inappropriate activation of amygdaloid neurons might contribute to misattribution of agency, wherein pathological activation of the amygdala would be misidentified as an externally generated voice, resulting in the auditory hallucinations. Consistent with the hypothesis, changes in the amygdala have been reported in human patients during auditory hallucinations (Dierks et al., 1999; Lennox et al., 2000). The auditory system and amygdala were more strongly activated by emotional words spoken to patients with auditory hallucinations than in patients in remission from auditory hallucinations as well as healthy controls (Escartí et al., 2010; Horga et al., 2014). Furthermore, reduction in the connectivity between the temporal lobe and the inferior frontal cortex in patients suffering from auditory hallucinations might induce reduction in feed forward signals to the temporal lobe, which would then reduce inhibition of the temporal lobe (i.e., disinhibition of the temporal lobe; Allen et al., 2012). In addition, external misattribution of distorted auditory feedback of self-generated vocalization was shown to be associated with activation of the temporal cortex (Fu et al., 2006). All of these findings are consistent with inappropriate activity of the auditory system and in particular the amygdala in patients with auditory hallucinations (Waters et al., 2012). Furthermore, amygdalar activation would facilitate prefrontal cortex activation of nucleus accumbens to induce positive schizophrenic symptoms (Grace, 2000). Interestingly, in humans, misattributions of self-generated speech to others are more prominent when the spoken word is emotionally valenced (Johns et al., 2001; Costafreda et al., 2008). Furthermore, in schizophrenia there are morphological and functional abnormalities in the amygdala and this may be related to distorted emotion perception in patients (Phillips et al., 2003). Thus, abnormal self/other vocalization selective activity of the amygdala and possibly elsewhere in the auditory system might be involved in auditory hallucinations. Further studies are required to test this hypothesis.

Dopamine mediated mechanism may be involved in the positive symptoms of schizophrenia including auditory hallucinations, as dopamine agonists and antagonists respectively increase and diminish the symptoms (Angrist et al., 1980). Interestingly, dopamine modulates auditory processing in the amygdala. Dopamine attenuates prefrontal cortical suppression of sensory inputs (Rosenkranz and Grace, 2001, 2002). In addition, dopaminergic activation enhances amygdalar responses to bottom-up thalamic inputs and suppresses amygdalar responses to cortical inputs (Chang and Grace, 2015). Taken together, these findings suggest that dopamine biases the amygdala toward the bottom-up signals, rather than more highly processed, top-down signals. This suggests that the bottom-up signals have less chance to be inhibited by the corollary discharges under dopaminergic activation, which may result in amygdalar activation in response to internally generated voices (i.e., auditory hallucinations). Future studies for recording Type-Other neurons in the amygdala under influences of dopaminergic agonists would be of interest to test this.

## Conclusion

By recording amygdalar neural responses to ultrasonic vocalization in male rats during social interaction, we showed the first neurophysiological evidence of differential responses to vocal affective social signals from self and others, and, in our population, the amygdalar neurons responded primarily to calls by others. The disturbance of self/other attribution in auditory hallucination in schizophrenia might be associated with dysfunctions of this type of responses in the amygdala. This experimental model could be further exploited for animal experiments about neural mechanisms of vocal communication, and its disturbance in psychiatric disorders including autism and schizophrenia (Konopka and Roberts, 2016).

## Author contributions

All authors listed, have made substantial, direct and intellectual contribution to the work, and approved it for publication.

## Funding

This work was supported partly by JSPS KAKENHI Grant Numbers 25830006 and 16H04652.

### Conflict of interest statement

The authors declare that the research was conducted in the absence of any commercial or financial relationships that could be construed as a potential conflict of interest.
