# Supplementary material for: Amygdalar Auditory Neurons Contribute to Self-Other Distinction during Ultrasonic Social Vocalization in Rats
Source: Front Neurosci. 2016 Sep 20;10:399. doi: 10.3389/fnins.2016.00399 (PMC5028407; doi:10.3389/fnins.2016.00399)
Supplement: Supplementary file 2 [file Image1.pdf]

Supplementary Material

Amygdalar auditory neurons contribute to self-other distinction during ultrasonic social vocalization in rats

Jumpei Matsumoto, Hiroshi Nishimaru, Yusaku Takamura, Susumu Urakawa, Taketoshi Ono, Hisao Nishijo*

*** Correspondence:** Dr. Hisao Nishijo, nishijo@med.u-toyama.ac.jp

1 Supplementary Data

Supplementary Movie 1. An example of ultrasonic vocalization during a social interaction between male rats. Ultrasound was converted into audible sound by dividing its frequency by 10.

2 Supplementary Figures

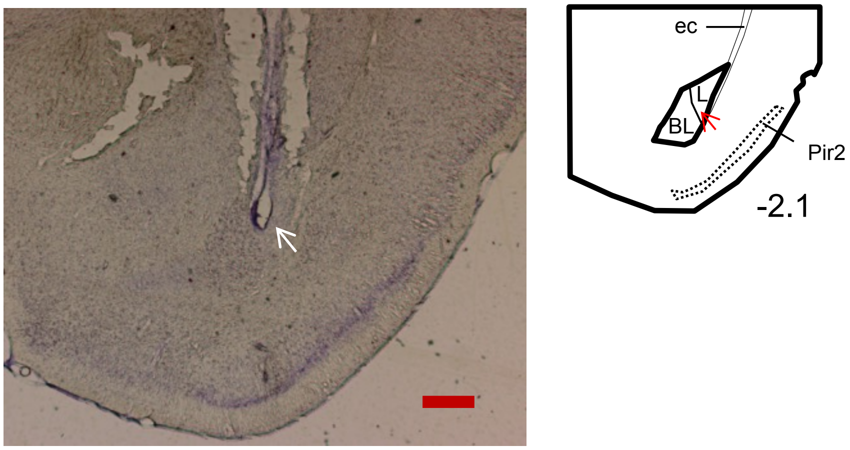

Supplementary Figure 1. An example of a coronal histological section with an electric lesion at the tip of tetrode (white arrow) in the lateral amygdaloid nucleus. Scale bar: 500 µm. The upper right inset shows the schematic of positions of anatomical structures. The red arrow corresponds to the white arrow in the photo. The value below indicates distance (mm) from bregma. L, lateral amygdaloid nucleus; BL, basolateral amygdaloid nucleus; Pir2, piriform cortex layer 2; ec, external capsule.

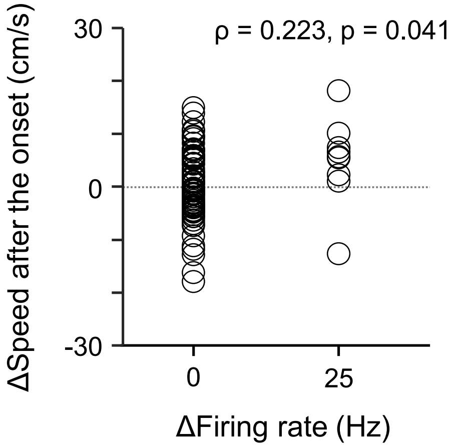

Supplementary Figure 2. A Type-Other neuron that showed significant correlations between neuronal activity and head movement (of the recorded rat) in response to the WFM calls by the conspecific. This is the same neuron as shown in Figure 3A. The scatter plot represents the relation between the change of the head movement speed after the onset of each call (ΔSpeed, see Figure 3G for the definition) and amygdalar neuronal response magnitude to each call [ΔFiring rate = (instantaneous firing rate in the bin that corresponded the peak in the averaged histogram, i.e., 0 to 40 ms from onset) – (instantaneous firing rate in the baseline bin, 80 to 40 ms prior to onset)]. Each open circle position represents ΔFiring rate and ΔSpeed associated with a single call. The two parameters were significantly correlated (Spearman's correlation analysis, $\rho = 0.223$, $p = 0.041$).
